# Risk of malignancy in ankylosing spondylitis: a systematic review and meta-analysis

**DOI:** 10.1038/srep32063

**Published:** 2016-08-18

**Authors:** Chuiwen Deng, Wenli Li, Yunyun Fei, Yongzhe Li, Fengchun Zhang

**Affiliations:** 1 Department of Rheumatology and Clinical Immunology, Peking Union Medical College Hospital, Chinese Academy of Medical Sciences & Peking Union Medical College, Key Laboratory of Rheumatology and Clinical Immunology, Ministry of Education, 1 Shuaifuyuan, Dongcheng District, 100005, Beijing, China; 2 Department of Rheumatology, China-Japan Friendship Hospital, Yinghua East Road, Chaoyang District, 100029, Beijing, China

## Abstract

Current knowledge about the overall and site-specific risk of malignancy associated with ankylosing spondylitis (AS) is inconsistent. We conducted a systematic review and meta-analysis to address this knowledge gap. Five databases (PubMed, EMBASE, Web of Science, the Cochrane library and the virtual health library) were systematically searched. A manual search of publications within the last 2 years in key journals in the field (Annals of the Rheumatic Diseases, Rheumatology and Arthritis & rheumatology) was also performed. STATA 11.2 software was used to conduct the meta-analysis. After screening, twenty-three studies, of different designs, were eligible for meta-analysis. AS is associated with a 14% (pooled RR 1.14; 95% CI 1.03–1.25) increase in the overall risk for malignancy. Compared to controls, patients with AS are at a specific increased risk for malignancy of the digestive system (pooled RR 1.20; 95% CI 1.01 to 1.42), multiple myelomas (pooled RR 1.92; 95% CI 1.37 to 3.69) and lymphomas (pooled RR 1.32; 95% CI 1.11 to 1.57). On subgroup analysis, evidence from high quality cohort studies indicated that AS patients from Asia are at highest risk for malignancy overall. Confirmation of findings from large-scale longitudinal studies is needed to identify specific risk factors and to evaluate treatment effects.

Ankylosing spondylitis (AS) is a chronic systemic inflammatory disease of the axial skeleton. Although the pathogenesis of AS remains to be fully elucidated, we do know that immune mechanisms play an important role in the pathophysiology of AS. Therefore, AS is thought to be an autoimmune disease that mainly affects males in the general population^1^. During the past decades, researchers have investigated the association between malignancy and autoimmune diseases, with various studies reporting an increased risk for cancer in patients with some autoimmune diseases^2^. As an example, rheumatoid arthritis (RA) is associated with a 28% increased risk for overall malignancy^3^. However, as the characteristics of AS are markedly different from those of RA, including anatomical distribution of affected joints, type of joint destruction, extra-articular manifestations and sex distribution, the positive association between RA and malignancy might not exist for patients with AS.

Several studies have evaluated the risk of malignancy associated with AS, with evidence being inconclusive^4^,^5^,^6^. Differences in risk across studies may be related to several factors, including: geographic region studies, study design and sample size, among other factors. Therefore, we undertook a systematic review and meta-analysis to derive a better estimation of the comparative risk of developing malignancy in AS patients versus the general population.

## Methods

The search was performed independently by two researchers (DCW and LWL), including screening of articles for eligibility and extraction of relevant data. Disagreements regarding eligibility of identified were resolved by consensus or by a third researcher (FYY).

### Search strategy

A literature search was conducted of the PubMed, EMBASE, Web of Science, Cochrane library, and Virtual Health Library databases using the following search terms: ankylosing spondylitis, cancer, malignancy, neoplasm, tumor, carcinoma, lymphoma, risk factor, odds ratio, relative ratio, hazard ratio, and standardized incidence rate. A representative search strategy of the PubMed database is provided in the supplementary files online. In addition, a specific manual search was also conducted of key journals of rheumatology (Annals of the Rheumatic Diseases, Rheumatology and Arthritis & Rheumatology) to identify relevant articles published within the two previous years. Manual search of the reference list of relevant studies identified in our search was also completed. Our search is up-to-date to May 2016.

### Eligibility criteria

The following criteria were included in our meta-analysis; (1) studies on human participants; (2) case-control studies, cohort studies and clinical trials; (3) those including medical history of AS as the exposure factor and malignancy as an outcome factor; (4) studies reporting estimated relative risk using relative risk (RR), standardized incidence ratio (SIR), hazard ratio (HR) or odds ratio (OR) of AS patients with malignancy; (5) the selection of cohort studies was made regardless of the specific AS management strategies used, and (6) those published in English. Studies were excluded based on the following criteria: (1) effect size could not be extracted or calculated; (2) in the case of duplicate publications, only one of them will be selected in our analysis, and (3) conference abstracts without subsequent publication in full text.

### Screening and data extraction

All articles identified by our search underwent a preliminary screening of their title and abstract to determine relevance and general adherence to eligibility criteria. Outcomes of our different searches were then merged, duplicate titles and publication removed. Full-text screening of retained titles was then performed to confirm eligibility.

The following data was extracted from retained studies: first author’s name; publication year; country in which the study was performed; study design; study period; and study results, including sex distribution, the number of AS patients with cancer, the overall risk of malignancy, controlled factors, and estimates of RR. Authors of the identified studies were contacted via e-mail if further details were needed.

### Evaluation of research quality

The research quality of retained studies was assessed using the Newcastle-Ottawa Scale (NOS). The NOS scoring system provides a maximum score of ‘9’ (stars) for 8 items, with the quality of study defined as follows: ≤3 stars, low quality; 4–6 stars, moderate quality; and ≥7 stars, high quality.

### Statistical analysis

Since the absolute risk of cancer is expected to be low overall, the four measures (SIRs, HRs and ORs) are expected to yield similar estimates to RR. Therefore, we transformed results from all studies into RR values to facilitate the subsequent meta-analysis^7^. Statistical analysis was performed using STATA 11.2 software (Stata Corporation, College Station, TX, USA). Heterogeneity was evaluated by the I^2^-statistic, with I^2^ < 25% considered as low heterogeneity, 25–50% as moderate, and >50% as a high degree of inconsistency. A pooled estimate of RR, and the corresponding 95% confidence interval (CI), was obtained using a random-effects or a fixed-effects model in the presence (I^2^ > 25%) or absence (I^2^ ≤ 25%) of heterogeneity, respectively. A P-value < 0.05 (two-sided) was considered significant. Subgroup analysis was performed for geographic region, study design, and location of malignancy by systems or organs. A sensitivity analysis was performed to evaluate stability by sequential omission of individual studies. To evaluate publication bias, we conducted a funnel plot and applied Begg’s test^8^.

## Results

### Characteristics of the included studies

Electronic and manual searches yielded 601 potentially eligible articles. A flow chart of the screening of articles for inclusion in our meta-analysis is shown in Supplementary Figure S1. During the first screening of titles and abstracts, 569 articles were excluded. Four additional studies were identified by manual search. A further 13 duplicate articles were excluded. All 23 eligible studies^4^,^5^,^6^,^9^,^10^,^11^,^12^,^13^,^14^,^15^,^16^,^17^,^18^,^19^,^20^,^21^,^22^,^23^,^24^,^25^,^26^,^27^,^28^, 17 cohort studies, 4 case-control studies, and 2 studies with *other* designs, were included in our meta-analysis (Table 1). Among these, 17 were from Europe, 3 from America, 2 from Asia, and 1 included population from several different regions. The number of reported cases of malignancy, and factors controlled for in estimating incidence and prevalence, are provided in Supplementary Table S1.

### Risk for malignancy associated with AS

Based on our meta-analysis, AS was associated with a 14% increase in the risk for overall malignancy (pooled RR, 1.14; 95% CI, 1.03–1.25; Table 2). In terms of the systems and/or organs involved, AS was associated with a specific higher risk for cancer of the digestive system (pooled RR, 1.20; 95% CI, 1.01–1.42) and malignant neoplasms of lymphoid, hematopoietic, and related tissue (pooled RR, 1.32; 95% CI, 1.11–1.57). As sufficient data on malignant neoplasms of lymphoid, hematopoietic, and related tissue was available, a targeted subgroup analysis was performed, indicating a relatively higher risk for patients with AS to develop multiple myelomas (pooled RR, 1.92; 95% CI, 1.37 to 2.69) and lymphomas (pooled RR, 1.32; 95% CI, 1.11 to 1.57; Table 2).

### Subgroup analysis

In the overall malignancy analysis, low heterogeneity was found (I^2^ = 37.8%). Therefore, we performed subgroup analyses by geographic region, study design, and NOS score to further explore factors influencing heterogeneity. A relatively higher risk for cancer was identified in patients with AS from Asia (pooled RR, 1.33; 95% CI, 1.15 to 1.54). However, cohort and high quality (NOS ≥ 7) studies also affected the heterogeneity (pooled RR, 1.15, 95% CI, 1.03 to 1.28; and pooled RR, 1.17, 95% CI, 1.05 to 1.30, respectively; Table 3).

### Publication bias and sensitivity analysis

Funnel plot and Begg’s test were used to evaluate publication bias. There was no obvious funnel plot asymmetry (Supplementary Figure S2). The P-value of Begg’s test was >0.05, indicative that publication bias was not evident in our meta-analysis. Our sensitivity analysis did not identify significant change in pooled RRs on sequential omission of individual studies, indicating that the results of this meta-analysis are stable and reliable.

## Discussion

Until now, the risk for malignancy associated with AS had not been fully clarified, with discrepancy in reported results. To our knowledge, this is the first systematic review and meta-analysis to assess the association between AS and malignancy. The majority of studies included in our meta-analysis were of high quality (15 studies with a NOS score ≥7), with 8 studies of lower quality (NOS score ≤6).

Our results indicate that AS is associated with an overall increased risk for malignancy (pooled RR, 1.14; 95% CI, 1.03–1.25), with overall low heterogeneity in results (Table 2). Subgroup analysis performed to explore the heterogeneity (Table 3) confirmed this association to be significant in Asian populations, but not in European or American populations. However, the overall number of patients with AS included in the subgroup analysis for Asian populations was small and, therefore, further research is needed to confirm an association between malignancy and AS in Asian populations. Secondly, the pooled RR for AS-associated malignancy was higher for cohort studies than for case-control studies and clinical trials. Therefore, study designs may be an influencing factor on the identified association between AS and malignancy. Therefore, our finding of a higher RR for specific cancers in patients with AS will need to be confirmed by clinical trials and case studies. Thirdly, about one quarter of selected articles for our meta-analysis were of moderate or low quality (NOS <7). These moderate-to-low quality studies did not identify a significant correlation between AS and malignancy, with high quality studies (NOS ≥7) reporting a significant association. Therefore, future studies will need to be well-designed to reduce the impact of study quality on the results.

An increased risk for malignant neoplasms of lymphoid, hematopoietic and related tissue (pooled RR 1.32; 95% CI, 1.11 to 1.57) was identified in patients with AS (Table 2), with the principal types being multiple myelomas (pooled RR 1.92; 95% CI, 1.37 to 1.69) and lymphomas (pooled RR 1.32; 95% CI, 1.11 to 1.57). Although prior studies have reported a correlation between AS and multiple myelomas and lymphomas, these correlations have typically been reported as part of a more general association between AS and malignancy. We conducted specific subanalyses that provide a more precise estimate on these associations.

Up to the final date of our search, May 2016, Hemminki *et al.*^12^ and Brown *et al.*^15^ reported that significantly elevated risks of MM were associated with broad categories of autoimmune diseases, including AS, while reported a specific elevated risk for multiple myelomas for broad categories of autoimmune diseases, including AS, while Lindqvist *et al.*^27^ did not find a specific association. Our meta-analysis, did identify a specific increased risk for multiple myelomas in patients with AS. However, our results do need to be further examined as the RR was calculated from a low number of studies. An increased risk for lymphomas was also reported in patients with AS. However, Hodgkin’s and non-Hodgkin’s lymphomas are generally grouped together and, therefore, the type of lymphomas associated with AS could not be specifically evaluated. Future studies should consider other grouping methods that would identify AS-associated risk for specific types of lymphomas. AS is also associated with an increased risk for cancers of the digestive system (pooled RR 1.20; 95% CI, 1.01 to 1.42), including liver, hepatobiliary tract and buccal cancer (Table 2). However, the association of AS to each of these specific cancers was only considered in one study and, therefore, further analysis of specific associations could not be conducted as part of our meta-analysis. Based on the outcomes of our meta-analysis, it would seem clinically reasonable to monitor patients with AS for the development of multiple myelomas, lymphomas, and cancer of the digestive system.

From a statistical perspective, the P-value is the primary output of statistical tests, and is used to differentiate a ‘true’ difference between variables and/or groups. Commonly, studies included in our meta-analysis used P < 0.05 as the threshold for declaration of statistical significance. However, some researchers have stated their concern regarding the misuse and misinterpretation of the 0.05 cutoff value, with no current consensus on alternative cutoffs having been reached^29^,^30^. In our meta-analysis, we did use the P-value for interpretation of specific results, such as in the evaluation of publication bias. So that we should be aware the difference found in this analysis might have different explanation when novel consensus of p-value is established.

The limitations of our meta-analysis need to be acknowledged. Foremost, although studies included in our meta-analysis focused on the risk for malignancy in patients with AS, studies included did vary in terms of their design and adjustment for covariates, which could be a potential source of bias of our results. As well, subgroup analysis could not be performed when reported cancers were not divided or described in details, limiting our ability to draw firm conclusions regarding AS as a risk factor for specific cancers. Although we did not identify an overall publication bias in our meta-analysis, we cannot rule out the potential bias contributed by smaller studies of moderate-to-low quality, which did not identify an increased risk for malignancy in patients with AS. It is also important to note that eligibility of studies was defined regardless of AS management strategies and, therefore, we cannot rule out possible effects of treatment on the risk of malignancy. Lastly, as with all systematic reviews, our search is limited by our institution’s access to databases. It was not possible to include Google Scholar (GS) in our search strategy, contrary to standard practice in systematic reviews. GS indexes many smaller journals that are not included in leading scholarly databases such as Pubmed. Studies published in GS-only journals may show smaller effects than other studies. Therefore, their absence from our review might lead our review to overstate the true magnitude of effect.

Despite these limitations, our meta-analysis provides new evidence of an increased risk for malignancy among patients with AS. Specific monitoring of patients with AS is recommended for early identification of cancer of digestive system, multiple myelomas, and lymphomas in clinical practice. Large-scale longitudinal studies will be essential to draw firm conclusions regarding AS-associated risk for malignancy, including effects of treatments and identification of disease- and person-specific risk factors. Recently, the British Society for Rheumatology Biologics Register in Ankylosing Spondylitis (BSRBR-AS) study has published their protocol for a prospective cohort study of the long-term safety and quality of life outcomes associated with biological treatments for AS (BSRBR-AS)^31^. This is a major ongoing study that will provide a standard for future studies evaluating the association between AS and malignancies, as well as identify future direction for research in AS.

## Additional Information

**How to cite this article**: Deng, C. *et al.* Risk of malignancy in ankylosing spondylitis: a systematic review and meta-analysis. *Sci. Rep.* **6**, 32063; doi: 10.1038/srep32063 (2016).

## Supplementary Material

Supplementary Information

## Figures and Tables

**Table 1:** Characteristics of selected studies on ankylosing spondylitis and risk of malignance.

Ref.	Study	Region	Design	Study period	Sex	AS patients	AS patients with cancer	Estimate of relative risk	NOS
[4]	Fallah *et al.*	Sweden	Cohort	1964–2010	F/M	17641	37	SIR	8
[5]	Hellgren *et al.*	Sweden	Cohort	2001–2010	F/M	8707	14	HR	8
[6]	Sun *et al.*	Taiwan	Cohort	1995–2010	F/M	4133	226	HR	7
[9]	van der *et al.*	America	Cohort	NA	F/M	1074	6	SIR	NA
[10]	Dreyer *et al.*	Danish	Cohort	2000–2008	F/M	861	8	SIR	6
[11]	Chang *et al.*	Korea	Cohort	2000–2012	F/M	NR	16	SIR	3
[12]	Hemminki *et al.*	Germany	Cohort	1964–2008	F/M	6646	16	SIR	7
[13]	Hemminki *et al.*	Germany	Cohort	1964–2008	F	1798	NR	SIR	7
[14]	Hemminki *et al.*	Germany	Cohort	1964–2008	F/M	5173	NR	SIR	6
[15]	Brown *et al.*	America	Cohort	1969–1996	F/M	NR	25	RR	7
[16]	Askling *et al.*	Sweden	Case-control	1964–2000	F/M	NR	23	RR	7
[17]	Feltelius *et al.*	Sweden	Cohort	1965–1995	F/M	6621	325	SIR	7
[18]	Becker *et al.*	Germany	Case-control	1999–2002	F/M	NR	3	OR	5
[19]	Anderson *et al.*	America	Case-control	1993–2002	F/M	NR	40	OR	8
[20]	Mellemkjaer *et al.*	Sweden	Case-control	1958–1998	F/M	NR	19	OR	8
[21]	Fallah *et al.*	Sweden	Cohort	1964–2010	F/M	NR	5	SIR	5
[22]	Castro *et al.*	Sweden	Cohort	1964–2008	F/M	NR	NR	SIR	8
[23]	Liu *et al.*	Sweden	Cohort	1964–2008	F/M	6646	NR	SIR	8
[24]	Carmona *et al.*	Spain	Cohort	1999–2005	F/M	761	16	SIR	7
[25]	Burmester *et al.*	Other regions	Clinical trails	NA	F/M	1684	NR	SIR	NA
[26]	Hellgren *et al.*	Sweden	Cohort	2001–2011	F/M	3078	53	RR	7
[27]	Lindqvist *et al.*	Sweden	Cohort	1965–2004	F/M	NR	12	OR	9
[28]	Hemminki *et al.*	Sweden	Cohort	1960–2012	F/M	17471	21	SIR	6

F: Female; M: Male; NR: Not reported; NA: Not applicable; Other regions: Europe, North America, South America, Asia, Australia, New Zealand and South Africa.

**Table 2:** Pooled relative risks of various malignance.

Malignancies	Number of studies	Pooled RR (95% CI)	Heterogeneity	
			I^2^(%)	p value
breast cancer	4	0.97 (0.79 to 1.20)	0.00	0.89
cancer of digestive system	5	1.20 (1.01 to 1.42)	12.30	0.33
cancer of urinary organs	5	1.14 (0.96 to 1.34)	14.80	0.31
cancer of male genital system	2	1.06 (0.82 to 1.35)	0.00	0.70
cancer of female genital system	3	1.12 (0.69 to 1.80)	37.60	0.16
endocrine cancer	2	1.42 (0.81 to 2.48)	0.00	0.63
malignant neoplasms of lymphoid, haematopoietic and related tissue	14	1.32 (1.11 to 1.57)	20.00	0.22
haematopoietic cancer	7	1.70 (1.38 to 2.09)	0.00	0.44
multiple myeloma	3	1.92 (1.37 to 2.69)	24.90	0.26
chronic lymphocytic leukaemia	2	2.13 (0.74 to 6.09)	0.00	0.62
not classified	2	1.40 (1.03 to 1.92)	0.00	0.59
lymphoma	9	1.32 (1.11 to 1.57)	0.00	0.98
Hodgkin’s lymphoma	2	1.37 (0.56 to 3.38)	0.00	0.82
non-Hodgkin’s lymphoma	5	1.03 (0.83 to 1.28)	0.00	0.87
not classified	3	0.97 (0.55 to 1.70)	0.00	0.64
respiratory cancer	3	1.31 (0.94 to 1.84)	29.80	0.24
skin cancer	3	0.72 (0.47 to 1.12)	0.00	0.87
cancers of other organs	4	1.22 (0.98 to 1.53)	7.20	0.38
overall cancer	23	1.14 (1.03 to 1.25)	37.80	0.02

**Table 3:** Statified analysis of pooled relative risks of cancer in ankylosing spondylitis.

Stratified analysis	Number of studies	Pooled RR (95% CI)	Heterogeneity	
			I^2^(%)	p Value
All				
Region				
European	17	1.07 (0.99 to 1.16)	8.10	0.35
Asia	2	1.33 (1.15 to 1.54)	0.00	0.46
America	3	1.56 (0.91 to 2.67)	71.10	0.03
Others	1	0.51 (0.19 to 1.39)	NA	NA
Design				
Cohort	17	1.15 (1.03 to 1.28)	45.80	0.00
Case-control	4	1.08 (0.83 to 1.41)	0.00	0.90
Pooled analysis from other studies	1	1.47 (0.60 to 3.58)	NA	NA
Clinical trails	1	0.51 (0.19 to 1.39)	NA	NA
NOS				
≥7	15	1.17 (1.05 to 1.30)	44.80	0.01
＜7	6	0.98 (0.77 to 1.26)	9.10	0.36
Others	2	0.89 (0.32 to 2.51)	58.20	0.12
