# Supplementary material for: Risk of malignancy in ankylosing spondylitis: a systematic review and meta-analysis
Source: Sci Rep. 2016 Aug 18;6:32063. doi: 10.1038/srep32063 (PMC4989281; doi:10.1038/srep32063)
Supplement: Supplementary Information [file srep32063-s1.pdf]

Risk of malignancy in ankylosing spondylitis: a systematic review and meta-analysis

Chuiwen Deng, MD,[1#], Wenli Li, PhD,[2#], Yunyun Fei, MD,[1*], Yongzhe Li, MD,[1], Fengchun Zhang, PhD,[1].

[1]Department of Rheumatology and Clinical Immunology, Peking Union Medical College Hospital, Chinese Academy of Medical Sciences & Peking Union Medical College, Key Laboratory of Rheumatology and Clinical Immunology, Ministry of Education, 100032, Beijing, China.

[2]Department of Rheumatology, China-Japan Friendship Hospital, Yinghua East Road, Chaoyang District, 100029, Beijing, China.

[#]These authors are the co-first authors on this study.

[*]Correspondence author: Yunyun Fei. Email address: feiyunyun2013@hotmail.com, Department of Rheumatology and Clinical Immunology, Peking Union Medical College Hospital, Peking Union Medical College and Chinese Academy of Medical Sciences, #41 Damucang xidan, Beijing, 100032, China. Tel: 86-10-69159966.

Supplementary Table S1 Other characteristics of selected studies.

Ref.	Study	Malignance	Controlled factor (adjustment for covariates)
[4]	Fallah et al.	non-Hodgkin lymphoma (37)	history of hospitalization for obesity and chronic obstructive pulmonary diseases
[5]	Hellgren et al.	lymphoma (14)	age, sex, and county of residence
[6]	Sun et al.	lung (36), liver (27), colorectal (31), prostrate (12), bladder (8), head and neck (24), female breast (18), corpus and uterus (5), haematopoietic (13), kidney (1), thyroid (14), other (47)	age, gender, hypertension, hyperlipidaemia, and diabetes
[9]	van der et al.	all,Non-melanoma skin carcinoma (2), other (4)	NA
[10]	Dreyer et al.	NR	various measures of disease activity at enrolment, gender, time since initiation, cumulative duration of treatment and age at

			treatment start
[11]	Chang et al.	all, liver cancer (3), lymphoid leukemia (2), thyroid cancer (2), other (9)	NR
[12]	Hemminki et al.	multiple myeloma	age, sex, period, region and socioeconomic status-specific standard incidence rates
[13]	Hemminki et al.	breast (59), cervix (7), endometrium (4), ovarian (8), other female genital (3)	age, sex, period, region and socioeconomic status-specific standard incidence rates
[14]	Hemminki et al.	upper digestive tract related cancer (14), esophageal adenocarcinoma (5), esophageal squamous cell carcinoma (2), stomach adenocarcinoma (13)	NR
[15]	Brown et al.	multiple myeloma	attained age, attained calendar year, latency between study entry and study exit, number of hospital visits and race.
[16]	Askling et al.	all, non-Hodgkin lymphoma (15), Hodgkin	time between the first

		lymphoma (2), chronic lymphocytic leukaemia(6)	discharge listing the ankylosing spondylitis and lymphoma diagnosis, age at first discharge listing ankylosing spondylitis, age at lymphoma diagnosis, decade of lymphoma diagnosis and sex
[17]	Feltelius et al.	all, buccal cancer (9), digestive organs (67), respiratory (34), breast (20), female genital system (11), prostate (55), testicular and other male cancer (3), kidney (16), urinary organs (20), melanoma skin cancer (9), Non-melanoma skin cancer (8), eye(2), nervous system (14), thyroid(2), endocrine(9), bone(2), connective tissue cancer (2), other(9), haematopoietic system(33)	sex, age at entry, attained age at cancer diagnosis, and follow up years, all yielded the same results. SIR for the overall risk was increased during first time period
[18]	Becker et al.	lymphoma	
[19]	Anderson et al.	non-Hodgkin lymphoma	age, gender, year of diagnosis/selection, race, and the number of physician

			claims
[20]	Mellemkjaer et al.	non-Hodgkin lymphoma	sex, age, year of diagnosis, and region
[21]	Fallah et al.	Hodgkin lymphoma	NR
[22]	Castro et al.	Hepatobiliary tract cancers (17), Primary liver cancer (10)	age, sex, period, region, socioeconomic status–specific person-years, smoking and for alcoholism
[23]	Liu et al.	prostate cancer (147), kidney cancer (25), bladder cancer (30)	age, sex, period, region, the socioeconomic status specific SIR, obesity, chronic obstructive pulmonary disease, smoking, and alcoholism
[24]	Carmona et al.	all, colon and rectum, lung, prostate, bladder, non-Hodgkin lymphoma, leukemia, detailed amount not reported	age, sex, duration of disease, disease severity at baseline, smoking, methotrexate and glucocorticoid use, and chronic obstructive

			pulmonary disease
[25]	Burmester et al.	all, lymphoma, non-melanoma skin cancer, detailed amount not reported	NA
[26]	Hellgren et al.	all, cancer of urinary organs (10), breast cancer (5), cancers of other organs(5)	age and sex
[27]	Lindqvist et al.	multiple myeloma	personal history of the condition, year of birth, year of diagnosis, sex, and county
[28]	Hemminki et al.	all, adenocarcinoma (10), squamous cell carcinoma (3), melanoma (2), undifferentiated (2)	NR

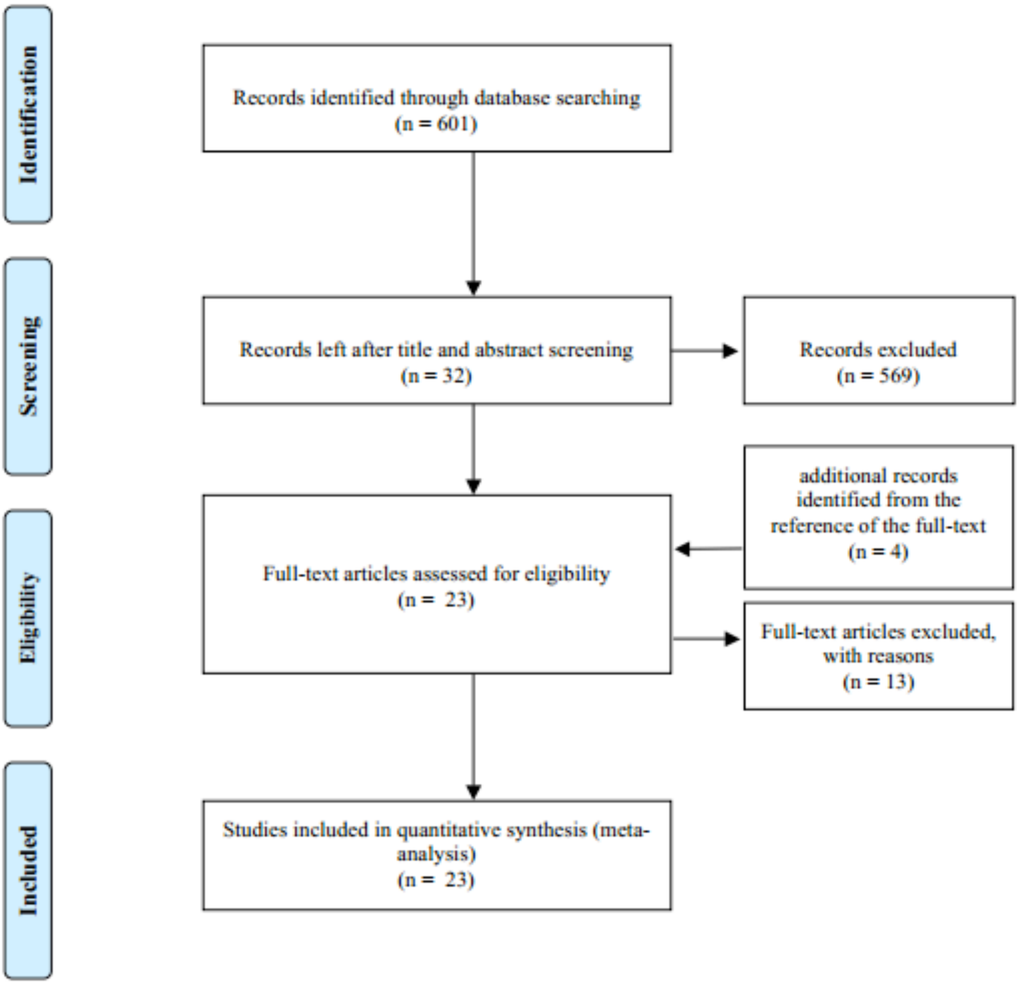

Supplementary Figure S1. Flow chart of studies included in the meta-analysis.

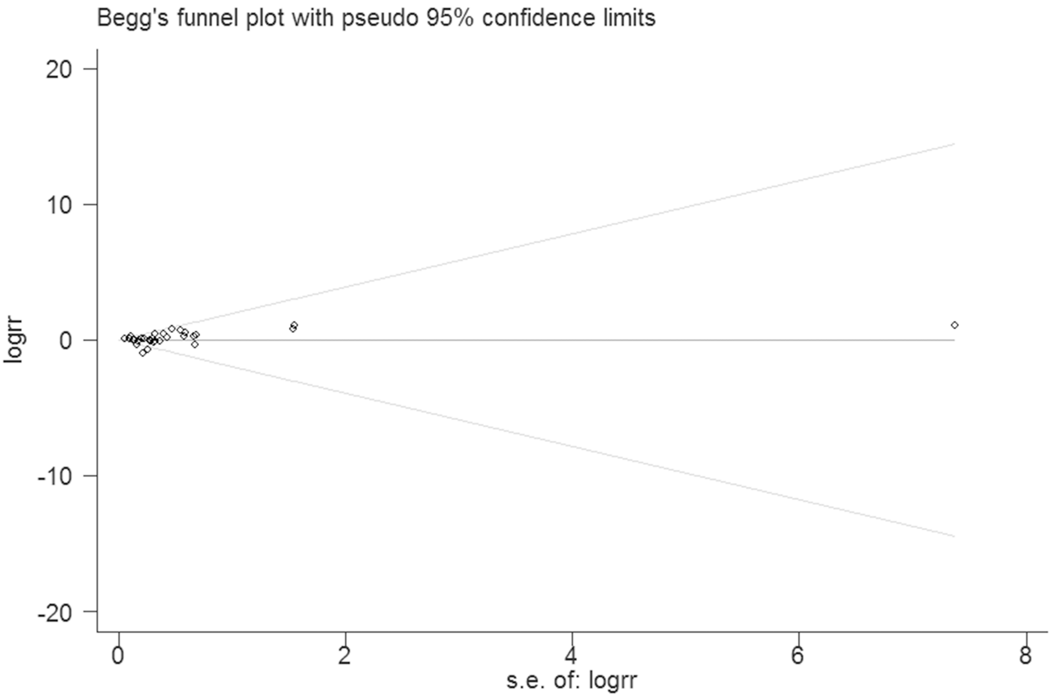

Supplementary Figure S2. Funnel plot of the studies focusing on overall cancer.

Search strategy of Pubmed

Key words: ankylosing spondylitis, cancer, malignancy, neoplasm, tumor, carcinoma, lymphoma, risk factor, odds ratio, relative ratio, hazard ratio, standardized incidence rate.

MeSH terms: related MeSH terms of the keywords were used.

Search details: ("spondylitis, ankylosing"[MeSH Terms] OR ("spondylitis"[All Fields] AND "ankylosing"[All Fields]) OR "ankylosing spondylitis"[All Fields] OR ("ankylosing"[All Fields] AND "spondylitis"[All Fields])) AND (("neoplasms"[MeSH Terms] OR "neoplasms"[All Fields] OR "cancer"[All Fields]) OR ("neoplasms"[MeSH Terms] OR "neoplasms"[All Fields] OR "malignancy"[All Fields]) OR ("neoplasms"[MeSH Terms] OR "neoplasms"[All Fields] OR "neoplasm"[All Fields]) OR ("tumour"[All Fields] OR "neoplasms"[MeSH Terms] OR "neoplasms"[All Fields] OR "tumor"[All Fields]) OR ("carcinoma"[MeSH Terms] OR "carcinoma"[All Fields]) OR ("lymphoma"[MeSH Terms] OR "lymphoma"[All Fields])) AND (("risk"[MeSH Terms] OR "risk"[All Fields] OR ("relative"[All Fields] AND "risk"[All Fields]) OR "relative risk"[All Fields]) OR RR[All Fields] OR (standardised[All Fields] AND ("epidemiology"[Subheading] OR "epidemiology"[All Fields] OR "incidence"[All Fields] OR "incidence"[MeSH Terms]) AND ("rate"[All Fields])) OR SIR[All Fields])
